# Water Filtration

**Published:** 1918-09

**Authors:** 


**A Monthly Review of Medicine and Surgery**

EDITOR AND PUBLISHER, DR. A. L. BENEDICT, 228 Summer St., corner of Elmwood Ave., Buffalo, (Address for all communications. Please make personal and telephone calls before 1 P. M.)

Third, in age, on the western continent. Independent, but supports professional organizations. News limited mainly to Western N. Y. and Penn.

Subscription $2.00 a year; $3.00 for two years in advance. Changes and errors in address or failure or duplication of delivery, should be reported immediately.

Yearly Volume 74 SEPTEMBER, 1918 Number 2

### Water Filtration.

We are all familiar with the kind of economy that has old clothes dyed and repaired at considerable expense and that secures something better than the original but which soon proves unsatisfactory and does not postpone except temporarily, the expense of a brand new outfit. Buffalo did something of this sort a few years ago when the water intake was put upstream. It has required no strictly expert knowledge or experience to show that the tremendous expense has not resulted in complete success from the sanitary standpoint. Anyone with a very moderate knowledge of local hydrography and sanitary science knew in advance that certain advantages would be secured, that a higher average of "purity" would be secured, either in the macroscopic or bacteriologic sense, that depreciation in quality from shore and creek contamination would be less marked and less frequent. It not only could have been but it was predicted with certainty that these advantages could not postpone the ultimate problem of securing a really safe water supply by filtration beyond the time when a thorough understanding of sanitary matters had become so general as to affect popular sentiment.

The joint problem of insuring an adequate water supply for the future and against an accident that might occur at any time, may be conceded to have been solved but it could have been solved by a much cheaper and shorter tunnel. Neither has the establishment of the new intake in any way expedited the construction of a filtration plant nor materially affected the economy of operation of such a plant when established.

However, it is sometimes wise to make the best of a bad bargain. In view of the enormous expense involved in a filtration plant, especially when we consider the financial problems of the present and the immediate future due to war, and the possibility that some system of sewage disposal other than the convenient one of dumping it into the water supply of down-stream communities may be forced upon our attention, it may be wise to stop short of ideal conditions.

We trust that our readers will not accuse us of ignorance of well known sanitary dicta, therefore, in presenting certain practical facts and in suggesting some possible ways of avoiding a very serious financial burden in the near future. First of all, it must be admitted that, no matter what it cost and whether proper forethought was exercised or not, Buffalo has an adequate and excellent water supply for all non-potable purposes and even for drinking and domestic purposes if we set the calendar back a few years. Even if the city should increase enormously in population and in commercial demand for water, the present water supply is susceptible of large increase at a relatively small expense as compared with a complete change of supply, and any such increase in demand would probably require a separation of potable and non-potable systems if prohibitive expense is to be avoided.

It is by no means certain that sand filtration is the last word in water sanitation. Chlorination has been employed satisfactorily by many communities and is, indeed, in some cases, mainly relied upon as a safeguard against bacterial contamination, even when clarification of mud and sand filtration are jointly practiced. It has proved tolerably satisfactory for Buffalo, as a temporary expedient. Various other methods of purification, at present only of academic interest, may almost instantly be put upon an economic basis on a large scale. If, for example, as some optimists already feel confident, electric or some other form of power becomes greatly reduced in expense, almost any water supply that would flow through pipes could be rendered safe, even if not aesthetically desirable, either by transformation into heat or along various lines already demonstrated theoretically.

Some general method of guarding the existing water supply in Lake Erie may become necessary for the mutual protection of other communities than our own and even a local application of this principle at our own expense might prove to be cheaper than some of the more elaborate devices proposed, notably sand filtration. Self purification of water in large volume and of scarcely appreciable current as in the case of Lake Erie, ''purifies itself,'' to use a somewhat old-fashioned sanitary term, within a comparatively few miles, largely by sedimentation and the action of harmless bacteria. Such means would not merely hold the sanitary problem stationary but would set it back, for practical purposes, to a period when the population of the water shed of the lake was comparatively sparse.

A very practical point to be considered is that Buffalo has, for some years, had a low typhoid rate notwithstanding the fact that fully half of the cases are introduced from without, either in the sense of being non-residents, or of having been contracted from extra-mural sources (vacational typhoid), or of being due to milk foods. When further allowance is made for infection by flies and dust into the outskirts of the city, for carriers and direct contact, the amount of typhoid that can be ascribed to our water supply or that could be ascribed to it even if chlorination were not practiced and if warnings to boil when the water becomes dangerous, were not published becomes very small though by no means negligible.

Furthermore, we must admit that the only water-borne disease that need be considered for practical purposes, is typhoid. Experience has accumulated to show that inoculation against this disease—as well as the paratyphoids—is almost if not quite as effective as against small pox though the test has not been intentionally carried on a large scale to the extent of deliberately exposing inoculated persons to highly contaminated water. But it is logical to ask whether routine inoculation, a comparatively cheap expedient would not obviate the necessity of elaborate treatment of water supplies especially as the protection would be general and not merely local.

For these several reasons, we believe that practical, if not theoretical wisdom points to a reasonable delay before adopting radical and enormously expensive methods in dealing with our water problem.

